# Retrospective In Silico Analysis of Routine Laboratory Data Supports a Specific Association of Epstein–Barr Virus and Multiple Sclerosis

**DOI:** 10.1111/ene.70430

**Published:** 2025-11-12

**Authors:** Mario Rodomonti, Florence Pache, Carolin Otto, Patrick Schindler, Bettina Eberspächer, Rohat Geran, Marco Puthenparampil, Paolo Gallo, Brigitte Wildemann, Sven Jarius, Hebun Erdur, Klemens Ruprecht

**Affiliations:** ^1^ Department of Neurology Charité—Universitätsmedizin Berlin, Corporate Member of Freie Universität Berlin and Humboldt‐Universität zu Berlin Berlin Germany; ^2^ Labor Berlin—Charité Vivantes GmbH Berlin Germany; ^3^ Department of Neuroscience University of Padua Padua Italy; ^4^ Division of Neuroimmunology, Department of Neurology University of Heidelberg Heidelberg Germany

**Keywords:** antibodies, cerebrospinal fluid, Epstein–Barr virus, multiple sclerosis, serum

## Abstract

**Background:**

We conducted a retrospective in silico analysis of routine laboratory data (RISAROLDA) to study the association of Epstein–Barr virus (EBV) and multiple sclerosis (MS).

**Methods:**

Patients with MS and 10 different inflammatory/neoplastic diseases were identified by ICD10 codes. Results of routine laboratory testing for antibodies to EBV, measles, mumps, rubella, herpes simplex virus, varicella zoster virus and cytomegalovirus were extracted using a digital tool.

**Results:**

Among 10,669 patients with MS and 42,222 controls, EBV serologies were available from 492 (4.6%) patients with MS and 1918 (4.5%) controls. While all but three patients with an ICD10 diagnosis of MS were EBV seropositive, closer inspection of the three EBV seronegative patients revealed they were misdiagnosed with MS, resulting in a 100% EBV seroprevalence in the remaining 489 patients with MS. In contrast, EBV seroprevalences were lower in all other diseases (78.6%–97.8%). Serum antibodies to the Epstein–Barr nuclear antigen‐1, but not to the viral capsid antigen, were higher in patients with MS than in all other diseases. In patients with MS, seroprevalences of all other common viruses were lower than those of EBV, but the frequency of intrathecal production of antibodies to EBV was lower than that of other common viruses.

**Conclusions:**

These findings suggest that the association of EBV and MS is specific for MS as compared to various other inflammatory/neoplastic diseases and that a negative EBV serology might be a marker for the absence of MS. RISAROLDA is a powerful approach for the screening of real‐world laboratory data.

## Graphical abstract

To study the association of Epstein–Barr virus (EBV) and multiple sclerosis (MS) as compared to 10 different control diseases, 10,669 patients with MS and 42,222 controls were identified by ICD10 codes and their available antiviral serologies retrieved from the hospital information system. EBV seroprevalence (Figure) and Epstein–Barr nuclear antigen‐1 antibodies were higher in MS than control diseases. Additionally, the seroprevalences and frequencies of an intrathecal production of antibodies to EBV and other common viruses were investigated in patients with MS.

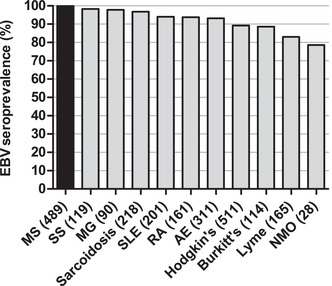

## 1 Introduction

Strong and consistent evidence suggests a causative link between infection with the Epstein–Barr virus (EBV) and multiple sclerosis (MS), a chronic inflammatory demyelinating disease of the CNS primarily affecting young adults [1, 2, 3]. Indeed, previous large seroepidemiological studies found a practically universal EBV seroprevalence in patients with MS (PwMS) and demonstrated that EBV infection precedes the clinical onset of MS [2, 4, 5, 6]. Furthermore, numerous case–control studies consistently observed higher serum levels of antibodies to the Epstein–Barr nuclear antigen‐1 (EBNA‐1) and, less consistently, to the EBV viral capsid antigen (VCA) in PwMS as compared to healthy controls [7, 8, 9, 10, 11, 12]. However, comprehensive analyses of the seroprevalence of EBV, as well as of EBNA‐1 and VCA serum antibody levels, in PwMS as compared to patients with other inflammatory neurological or systemic diseases, or patients with EBV‐associated cancers, have hitherto not been conducted. Likewise, large and systematic studies comparing the seroprevalence of EBV with that of other common viruses in PwMS are scarce.

‘Big Data’ techniques are a powerful tool for mining digitally stored data from high numbers of patients [13]. In particular, analyses of digitally stored laboratory data obtained during routine clinical care allow for the retrospective study of results from laboratory diagnostics, including determinations of antiviral antibodies, in very large numbers of patients with different diseases. This approach is based on the concept that even if only a fraction of all patients included in a specific screening underwent a given laboratory test, the very large number of patients included in the screening will still result in high numbers of patients who have undergone that laboratory test and these patients may be considered as a sample representative of the source population.

Here, we systematically analysed the seroprevalence of EBV, as well as serum levels of antibodies to EBNA‐1 and VCA, in PwMS compared to patients with 10 different control diseases by an in silico analysis of laboratory data obtained during routine clinical care. We also used this approach, which we term retrospective in silico analysis of routine laboratory data (RISAROLDA), to compare the seroprevalences of EBV and other common viruses and the frequencies of intrathecal antibody production against EBV and other common viruses in PwMS.

## 2 Patients and Methods

### 2.1 Patient Identification

The study was approved by the ethical committee of Charité—Universitätsmedizin Berlin, Berlin, Germany (EA2/009/25). The medical diagnoses of all inpatients and outpatients treated at Charité—Universitätsmedizin Berlin are documented by ICD10 codes in the hospital information system (i.s.h.med, SAP, Walldorf, Germany) of Charité—Universitätsmedizin Berlin. This system offers a tool for searching patients according to their ICD10 diagnoses, whose output is lists of patients, as identified by a patient number, which is unique for each patient, and a case number, which is unique for each admission to the hospital. Additionally, the lists include the respective ICD10 diagnosis, as well as the patients' date of birth. We used this tool to compile lists of all patients treated at Charité—Universitätsmedizin Berlin between 1 January 2000 and 30 November 2022 with ICD10 diagnoses of multiple sclerosis (G35.0, G35.1, G35.2, G35.3, G35.9), Sjogren's syndrome (M35.0), myasthenia gravis (G70.0), sarcoidosis (D86.0, D86.1, D86.2, D86.3, D86.8 and D86.9), systemic lupus erythematosus (M32.0, M32.1, M32.8, M32.9), rheumatoid arthritis (M06.0, M06.1, M06.2, M06.3, M06.4, M06.8, M06.9), autoimmune encephalitides (G04.8), Hodgkin's lymphoma (C81.0, C81.1, C81.2, C81.3, C81.4, C81.7, C81.9), Burkitt's lymphoma (C83.7), Lyme disease/borreliosis (A69.2) and neuromyelitis optica spectrum disorders (NMOSD, G36.0) (see also Table 1). In patients who were treated more than once at Charité—Universitätsmedizin Berlin during the study period, we only considered results from the first admission.

**TABLE 1:** ICD10 codes, total number of patients, patients with available EBV serologies, rate of EBV seropositivity and age of patients with multiple sclerosis and 10 different control diseases.

Diagnosis	ICD10 code	Number	EBV serology available, *n* (%)	EBV seropositive, *n* (%)	Age, mean (±SD), years	Age, median (min–max), years
Multiple sclerosis						
Initial manifestation of MS	G35.0	1610	144 (8.9)	142 (98.6)^a^	36.4 (12.7)	35 (7–71)
Relapsing–remitting MS	G35.1	4649	202 (4.3)	202 (100)	35.4 (12.8)	33 (9–74)
Primary progressive MS	G35.2	990	50 (5.1)	50 (100)	44.3 (13.5)	42.5 (12–73)
Secondary progressive MS	G35.3	1310	55 (4.2)	55 (100)	45.4 (11.8)	47 (22–79)
MS, unspecified	G35.9	2110	41 (1.9)	40 (97.6)^a^	36.2 (16.3)	33 (12–69)
Sum	—	10,669	492 (4.6)	489 (99.4)	—	—
Control diseases						
Sjogren's syndrome	M35.0	5275	119 (2.3)	117 (98.3)	48.1 (16.6)	50 (6–80)
Myasthenia gravis	G70.0	3871	90 (2.3)	88 (97.8)	54 (20.7)	57.5 (0–83)
Sarcoidosis	D86^b^	4161	218 (5.2)	211 (96.8)	44.7 (16.6)	46 (1–81)
Systemic lupus erythematosus	M32^c^	3837	201 (5.2)	189 (94)	37.6 (16.6)	37 (4–89)
Rheumatoid arthritis	M06^d^	13,028	161 (1.2)	151 (93.8)	51.4 (20.5)	56 (3–88)
Autoimmune encephalitides	G04.8	3448	311 (9)	290 (93.2)	39.4 (21.7)	38 (0–86)
Hodgkin's lymphoma	C81^e^	2578	511 (19.8)	456 (89.2)	35.2 (21.3)	30 (1–86)
Burkitt's lymphoma	C83.7	2850	114 (4.0)	101 (88.6)	29.9 (21.4)	27 (1–84)
Lyme disease	A69.2	2821	165 (5.8)	137 (83)	36 (22.1)	38 (2–91)
Neuromyelitis optica	G36.0	353	28 (7.9)	22 (78.6)	43.9 (20.2)	47 (1–80)
Sum	—	42,222	1918 (4.5)	1762 (91.9)	—	—

Abbreviations: EBV = Epstein–Barr virus, ICD10 = International Classification of Diseases, tenth revision, MS = multiple sclerosis, SD = standard deviation.

^a^ Note that in the two EBV seronegative patients with an ICD10 diagnosis of initial manifestation of MS and the one EBV seronegative patient with a diagnosis of unspecified MS, diagnoses were not correct (see also main text and Table 2).

^b^ Includes D86.0, D86.1, D86.2, D86.3, D86.8 and D86.9.

^c^ Includes M32.0, M32.1, M32.8, M32.9.

^d^ Includes M06.0, M06.1, M06.2, M06.3, M06.4, M06.8 and M06.9.

^e^ Includes C81.0, C81.1, C81.2, C81.3, C81.4, C81.7, C81.9.

### 2.2 Identification of Laboratory Data

Laboratory data generated at the laboratories serving Charité—Universitätsmedizin Berlin are digitally stored in the hospital information system of Charité—Universitätsmedizin Berlin. This system has a digital tool for searching for laboratory data, which allows identifying results of specific laboratory tests based on the patients' case numbers and specific laboratory test identification numbers. We thus entered lists of case numbers generated during patient identification (see above) in this tool to search for results of antiviral antibody determinations. The various assays used for the determination of antiviral antibodies are listed in Table S1.

Patients were considered EBV seropositive if EBNA‐1 IgG or VCA IgG or VCA IgM was positive. Patients were considered EBV seronegative if EBNA‐1 and VCA IgG and VCA IgM were negative. Patients were considered seropositive for rubella virus, measles virus, mumps virus, herpes simplex virus (HSV), varicella zoster virus (VZV) and cytomegalovirus (CMV) if IgG for the respective viruses was positive.

The patients' age at the time of laboratory testing was calculated from the date of the serological test and the date of birth. Clinical data of three EBV seronegative patients with an ICD10 diagnosis of MS were retrieved from the patients' medical records.

### 2.3 EBNA‐1 and VCA Serum Antibody Levels in Patients With MS and Controls

From a subgroup of PwMS and control diseases, quantitative data on serum levels of antibodies to EBNA‐1 and VCA (in U/mL) as measured by Liaison (DiaSorin, Saluggia, Italy) automated quantitative chemiluminescence immunoassay (CLIA) at Labor Berlin GmbH, Berlin, Germany, were available. The assay range of the EBNA‐1 IgG CLIA is 3 to 600 U/mL and of the VCA IgG CLIA 10 to 750 U/mL. EBNA‐1 IgG levels < 5 U/mL and VCA IgG levels < 20 U/mL were considered negative. EBNA‐1 IgG levels > 600 U/mL were set to 600 U/mL and VCA IgG levels > 750 U/mL were set to 750 U/mL.

### 2.4 Intrathecal Antiviral Antibody Production

In the group of PwMS, data from routine diagnostic testing for intrathecal production of antiviral antibodies were likewise extracted from the hospital information system. Intrathecal antiviral antibody production was assessed by calculating antibody indices (AIs) according to a standard formula [14]. AI determinations were performed by measuring EBV IgG, rubella virus IgG, measles virus IgG, mumps virus IgG, HSV IgG, VZV IgG and CMV IgG in cerebrospinal fluid (CSF) and serum samples of PwMS. The various assays used for the determination of antiviral AIs are listed in Table S1. AI values ≤ 1.5 were considered normal. AI values > 1.5 were considered elevated, indicating an intrathecal production of antiviral antibodies.

### 2.5 Statistical Analyses

For descriptive statistics, categorical data are reported as absolute and relative (%) frequencies and continuous data as mean (standard deviation) or median (absolute range). The statistical significance of differences in the seroprevalence of EBV in PwMS as compared to patients with other diagnoses was assessed by Chi‐square tests. The association of EBV seroprevalence with age was assessed by Spearman rank correlation. Statistical significance of differences between EBNA1 or VCA antibody titers in PwMS and controls was assessed by Mann–Whitney tests. All statistical analyses were conducted using R version 4.2.0 in RStudio. *p* values < 0.05 were considered significant. Due to the exploratory nature of this study no correction for multiple testing was performed.

## 3 Results

### 3.1 Higher EBV Seroprevalence in Patients With MS Than in 10 Different Control Patient Groups

To compare the EBV seroprevalence in PwMS with that of patients with other diagnoses we digitally searched for all patients with an ICD10 diagnosis of MS treated at Charité—Universitätsmedizin Berlin between 1 January 2000 and 30 November 2022. This identified a total of 10,669 patients (Table 1). As expected, the median age of patients with primary progressive MS (PPMS, 42.5 years) and secondary progressive MS (SPMS, 47 years) was higher than that of patients with a first clinical manifestation of MS (35 years) and relapsing remitting MS (RRMS, 33 years). Next, we similarly identified patients with immune‐mediated neurological diseases other than MS (myasthenia gravis, autoimmune encephalitides, neuromyelitis optica spectrum disorders), patients with systemic/rheumatologic inflammatory diseases (Sjogren's syndrome, sarcoidosis, systemic lupus erythematosus, rheumatoid arthritis, Lyme disease) as well as patients with EBV‐associated neoplastic diseases (Hodgkin's lymphoma, Burkitt's lymphoma). This resulted in a total of 42,222 control patients, with group sizes varying between 353 (neuromyelitis optica spectrum disorders) and 13,028 (rheumatoid arthritis). A digital search for results of EBV serologies identified available EBV serology results in a similar percentage of PwMS (*n* = 492, 4.6%) and control patients (*n* = 1918, 4.5%). While all patients with ICD10 diagnoses of RRMS, PPMS and SPMS were EBV seropositive, two patients with an ICD10 diagnosis of first clinical manifestation of MS (G35.0) and one patient with an ICD10 diagnosis of G35.9 (MS, unspecified) were EBV seronegative, resulting in an overall EBV seroprevalence of 489/492 (99.4%) in the MS group.

However, given the previously described practically universal EBV seroprevalence in MS, the 3 EBV seronegative patients with an ICD10 diagnosis of MS appeared conspicuous [4, 5, 6]. Indeed, closer inspection showed that MRI and CSF findings of these 3 patients were not suggestive of MS and none of them met current diagnostic criteria for MS (Table 2) [15]. We therefore conclude that these patients did not have MS and that the ICD10 diagnoses of these 3 patients deposited in the hospital information system were not correct. Consequently, all (100%) of the remaining 489 PwMS were EBV seropositive.

**TABLE 2:** Clinical, magnetic resonance imaging (MRI) and cerebrospinal fluid (CSF) findings of 3 EBV seronegative patients with a false ICD10 diagnosis of MS.

	Patient #1	Patient #2	Patient #3
ICD diagnosis	G35.9	G35.0	G35.0
Age at diagnosis, years	38	7	16
Clinical presentation	Speech disorder, dizziness, headaches, long‐standing distal discomfort of the legs	Paroxysmal weakness of legs, paroxysmal paresthesia of the upper and lower extremities	Visual impairment in the right eye, suspected optic neuritis
MRI findings	Cranial MRI: inconspicuous findings, no evidence of chronic inflammatory CNS disease, multiple subcutaneous masses due to Proteus syndrome	Cranial MRI: inconspicuous findings, no evidence of demyelinating disease; spinal MRI: inconspicuous findings	Cranial MRI: no evidence of chronic inflammatory CNS disease; unspecific diffuse right parietal hyperintensity not typical for demyelination; spinal MRI: normal
CSF findings	1 white cell/μL, no oligoclonal bands in CSF and serum, no intrathecal immunoglobulin synthesis	0.33 white cells/μL; no oligoclonal bands in CSF and serum	2 white cells/μL; no oligoclonal bands in CSF and serum
Diagnostic criteria for MS (McDonald 2017) fulfilled	No	No	No

**FIGURE 1:**
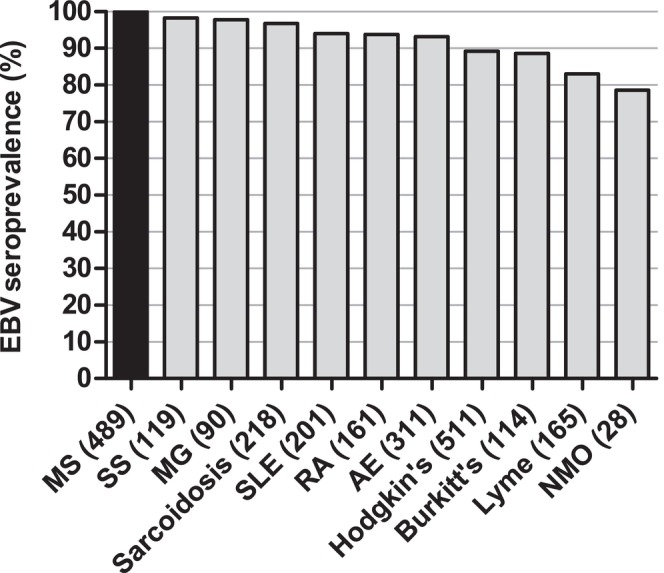
EBV seroprevalences in patients with MS and patients with 10 different control diseases. The number of patients with a given diagnosis is indicated in parentheses. Statistical significance of differences of the EBV seroprevalences between patients with MS and patients with other diseases was assessed by Chi‐square tests, demonstrating significant differences in all pairwise comparisons (*p* < 0.05). AE = autoimmune encephalitides, MG = myasthenia gravis, MS = multiple sclerosis, NMO = neuromyelitis optica, RA = rheumatoid arthritis, SLE = systemic lupus erythematosus, SS = Sjogren's syndrome.

EBV seroprevalences in the 10 different control disease groups were lower than the 99.4% (489/492) EBV seroprevalence in the MS group, ranging between 78.6% (NMOSD) and 98.3% (Sjogren's syndrome) (Table 1, Figure 1). In pairwise comparisons, the lower EBV seroprevalences in the control disease groups as compared to the 99.4% (489/492) EBV seroprevalence in the MS group achieved statistical significance (*p* < 0.05) for all control disease groups, except for the groups of patients with Sjogren's syndrome (*p* = 0.25) and myasthenia gravis (*p* = 0.13). Likewise, when compared to the 99.4% (489/492) EBV seroprevalence in the MS group, the EBV seroprevalence of all control patients combined (1762/1918, 91.9%, *p* < 0.0001) was significantly lower than that of the MS group.

**FIGURE 2:**
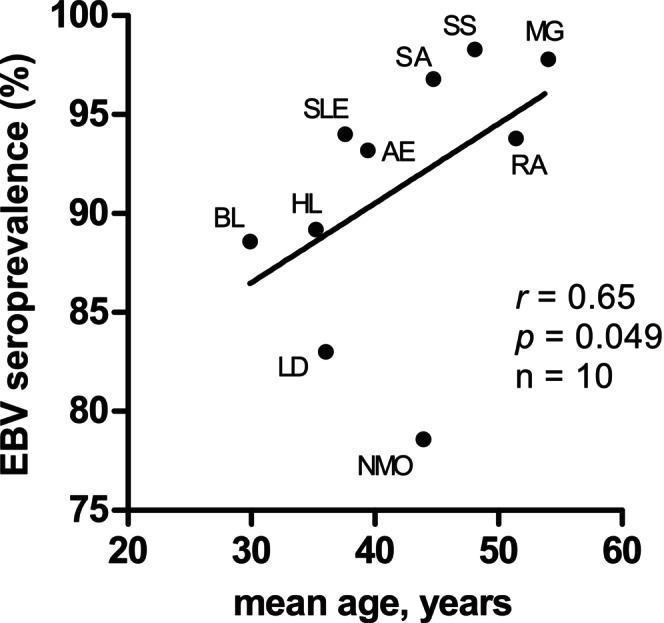
Correlation of EBV seroprevalence rates with the mean age of patients in the 10 different control disease groups. The significance of the association of EBV seroprevalences with age was assessed by Spearman rank correlation. AE = autoimmune encephalitides, BL = Burkitt's lymphoma, HL = Hodgkin's lymphoma, LD = Lyme disease, MG = myasthenia gravis, NMO = neuromyelitis optica, *r* = Spearman's *r*, RA = rheumatoid arthritis, SA = sarcoidosis, SLE = systemic lupus erythematosus, SS = Sjogren's syndrome.

As EBV seroprevalence increases with increasing age, we wondered whether the different EBV seroprevalence rates in the 10 control patient groups might be related to the patients' age. Indeed, EBV seroprevalence rates among the 10 different control groups correlated with the mean age of the different disease groups (*r* = 0.65, *p* = 0.049, Figure 2). A notable outlier was patients with NMOSD who had a relatively low EBV seroprevalence (78.6%) at a mean age of 43.9 years. However, as EBV serologies were available from only 28 patients with NMOSD, this group should be regarded with caution. Omission of the NMOSD group from the analysis shown in Figure 2 resulted in an even stronger correlation of EBV seroprevalence with age (*r* = 0.78, *p* = 0.017).

Plotting of the EBV seroprevalences in the MS (*n* = 492), Hodgkin's lymphoma (*n* = 511) and autoimmune encephalitides (*n* = 311) groups binned into 10‐year age intervals demonstrated an increase of EBV seroprevalences with age in the Hodgkin's lymphoma and autoimmune encephalitides groups. In contrast, EBV seroprevalence was already high at a young age and remained continuously high throughout all age intervals in the MS group (Figure S1).

### 3.2 Higher EBNA‐1 Antibody Levels in Patients With MS Than in Control Patient Groups

**FIGURE 3:**
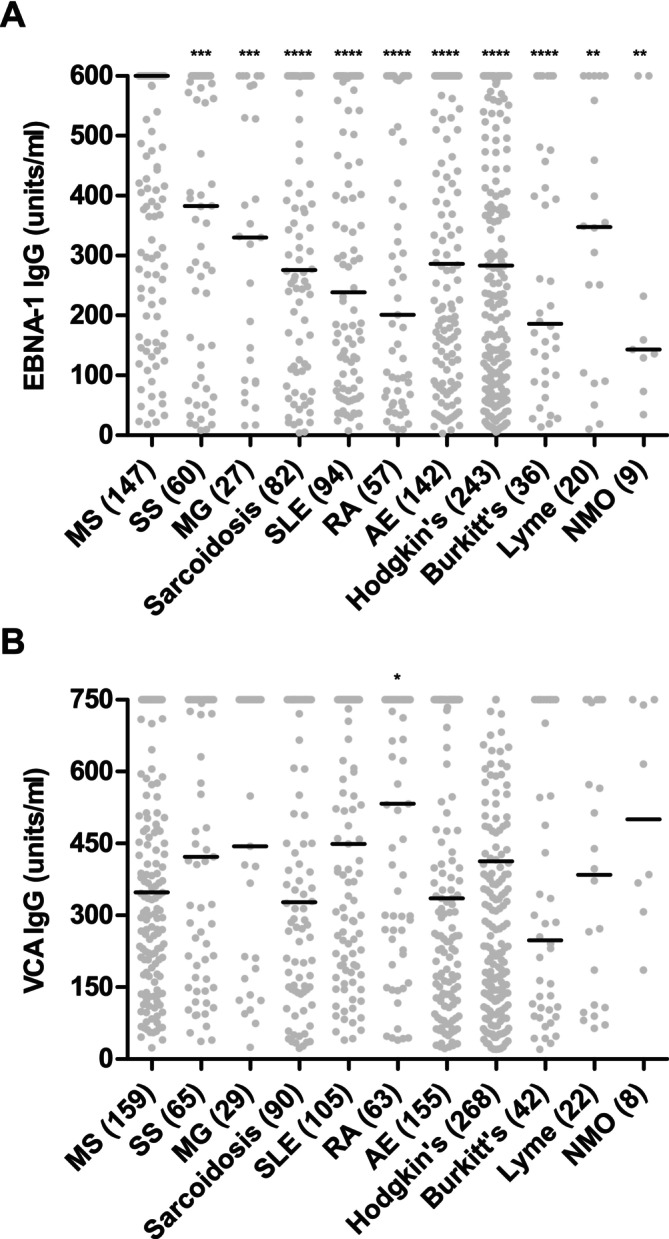
Levels of EBNA‐1 IgG (A) and VCA IgG (B) in sera of patients with MS and patients with 10 other diseases. The number of patients with a given diagnosis is indicated in parentheses. Statistical significance of differences between patients with MS and patient with other diseases was assessed by Mann Whitney tests. Thick horizontal lines indicate the median. *p < 0.05, **p < 0.01, ***p < 0.001, ****p < 0.0001. AE = autoimmune encephalitides, EBNA‐1 = Epstein–Barr nuclear antigen‐1, IgG = immunoglobulin G, MG = myasthenia gravis, MS = multiple sclerosis, NMO = neuromyelitis optica, RA = rheumatoid arthritis, SS = Sjogren's syndrome, VCA = viral capsid antigen.

In a proportion of PwMS and control patients with other diseases, quantitative data on serum levels of EBNA‐1 and VCA antibodies were available. As shown in Figure 3A, serum levels of EBNA‐1 antibodies were clearly higher in PwMS than in each of the 10 control patient groups (*p* ≤ 0.01 for all pairwise comparisons). In contrast, serum levels of antibodies to VCA did not differ in PwMS and the control patient groups (Figure 3B).

### 3.3 Higher Seroprevalences of EBV Compared to Other Common Viruses in Patients With MS

**FIGURE 4:**
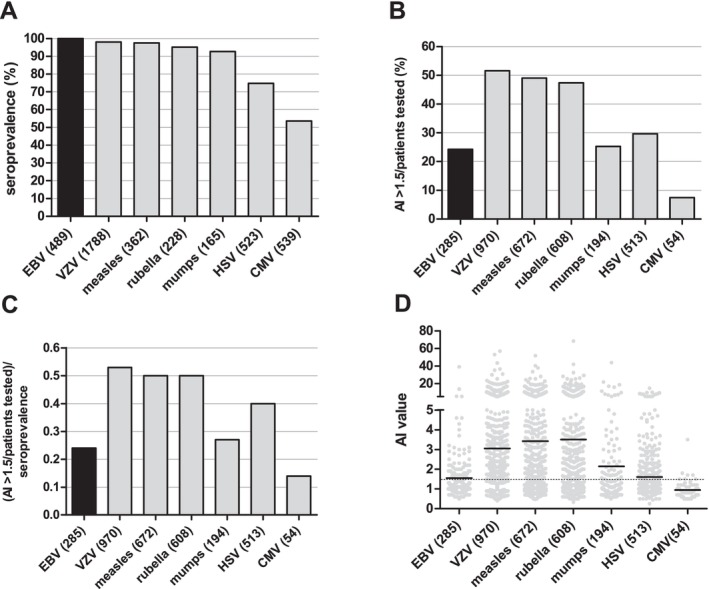
(A) Seroprevalences of EBV and six other common viruses in patients with MS. The number of patients with results of antiviral antibody tests available is indicated in parentheses. (B) Frequencies of elevated AIs (AI > 1.5) to EBV and six other common viruses among patients with MS in whom AIs to the respective viruses were determined. The number of patients with results of AI determinations available is indicated in parentheses. (C) Index indicating the frequencies of an elevated antiviral AI among patients with MS with available results of AI determinations divided through the seroprevalence of the respective virus in patients with MS. The number of patients included in the analyses is indicated in parentheses. (D) Absolute AI values for EBV and 6 further microbes in patients with MS. The number of patients with available data is indicated in parentheses. Thick horizontal lines indicate the mean. The dashed line indicates the cut‐off of 1.5, AI levels above which were considered elevated. AI = antibody index, CMV = cytomegalovirus, EBV = Epstein–Barr virus, HSV = herpes simplex virus, VZV = varicella zoster virus.

We also used the RISAROLDA approach to compare the seroprevalence of EBV to that of other common viruses, that is, measles, rubella and mumps virus, VZV, HSV and CMV, in PwMS. Results of determinations of non‐EBV antiviral antibodies were available from a total of 3605 PwMS with group sizes ranging from *n* = 165 (mumps virus) to *n* = 1788 (VZV) (Table 3). Seroprevalences of antibodies to these viruses ranged between 53.6% (CMV) and 98% (VZV) and were thus lower than the 100% seroprevalence of EBV in PwMS (Figure 4A).

**TABLE 3:** Seroprevalences of EBV and other common viruses and frequencies of intrathecal antibody production to EBV and other common viruses in patients with MS (*n* = 10,669).

Virus	Serology available, *n*	Seropositive, *n* (%)	Results of AI determinations available, *n*	AI > 1.5 among patients with results of AI determinations available, *n* (%)	Percentage of patients with AI > 1.5/percentage of seropositive patients, index
EBV	489	489 (100)	285	69 (24.2)	0.24
VZV	1788	1753 (98)	970	500 (51.6)	0.53
Measles virus	362	353 (97.5)	672	329 (49.0)	0.50
Rubella virus	228	217 (95.2)	608	288 (47.4)	0.50
Mumps virus	165	153 (92.7)	194	49 (25.3)	0.27
HSV	523	391 (74.8)	513	152 (29.6)	0.40
CMV	539	289 (53.6)	54	4 (7.4)	0.14

Abbreviations: AI = antibody index, CMV = cytomegalovirus, EBV = Epstein–Barr virus, HSV = herpes simplex virus, VZV = varicella zoster virus.

### 3.4 Low Frequency of Intrathecally Produced EBV Antibodies as Compared to Antibodies to Other Common Viruses in Patients With MS

More than 90% of PwMS have an intrathecal production of IgG, which is typically detected by the presence of CSF specific oligoclonal bands [16]. It is well known that the intrathecally produced IgG is polyspecific and partly directed against common viruses [17]. The intrathecal production of antiviral IgG can be assessed by calculating antibody indices (AI) according to standard formula [14]. While AIs normally range between 0.7 and 1.3, an AI > 1.5 is considered to indicate an intrathecal production of antiviral IgG. We used the RISAROLDA approach to identify results of AI determination in PwMS and to compare the frequency of elevated EBV AIs to the frequencies of elevated AIs against other common viruses (Table 3). Results of EBV AI determinations were available from 285 PwMS. Results of non‐EBV AI determinations were available from 3011 PwMS with group sizes ranging from *n* = 54 (CMV) to *n* = 970 (VZV). Frequencies of elevated AIs for measles, rubella and VZV were roughly 50% and thus about twice as high as the frequency of elevated EBV AIs (24.2%) (Figure 4B). We previously showed that elevated AIs do practically not occur in patients who are seronegative to a given virus [18]. Accordingly, the frequency of an elevated AI to a given virus should preferentially be determined only in those patients who are seropositive to that virus [18]. However, the data obtained by the RISAROLDA approach did not permit establishing the seroprevalences of the respective viruses in the same patients of whom AI determinations were available. Nevertheless, to relate the frequencies of elevated AIs to the seroprevalence of the respective viruses we divided the ‘raw’ frequency of an elevated AI (in %) by the previously established seroprevalence of the respective virus in PwMS (see Table 3, Figure 4A). The resulting index showed that, except for CMV, the seroprevalence corrected AI frequency was lower for EBV than for all other investigated viruses (Figure 4C). Also, the absolute EBV AI values were low when compared to measles, rubella and VZV AIs (Figure 4D).

## 4 Discussion

The key findings of this analysis of the association of EBV and MS by a ‘Big Data’ approach are (i) EBV seroprevalences in 10 different control disease groups do not reach the 100% EBV seroprevalence observed in PwMS, (ii) a 100% EBV seroprevalence was detected in patients with RRMS, PPMS and SPMS, (iii) a negative EBV serology may be a marker for the absence of MS, (iv) serum levels of antibodies to EBNA‐1, but not VCA, are higher in PwMS than in 10 different control diseases, (v) seroprevalences of 6 other common viruses are lower than that of EBV in PwMS, (vi) yet, intrathecal EBV antibody production is lower than that of other common viruses in PwMS and (vii) RISAROLDA is an efficient and powerful approach for screening of results from laboratory data in large groups of patients.

The practically universal EBV seroprevalence observed in this work is consistent with previous results from others and our group and confirms the strong association of EBV and MS [2, 4, 6]. However, as a novel contribution, the digital screening applied in this study permitted us to establish EBV seroprevalences in large numbers of patients with 10 further disease entities, including inflammatory and neoplastic diseases that have previously been associated with EBV. In none of these diseases, EBV seroprevalence was as high as in PwMS. Rather, the absolute EBV seroprevalences and the increasing EBV seroprevalence with increasing age in the control disease groups are in accordance with previously reported EBV seroprevalences in the general population and large hospital populations [6, 19, 20]. Altogether, while we cannot fully exclude that there may be other diseases with a similarly strong EBV association than MS, our present data suggest that a practically universal EBV seroprevalence is a rather specific characteristic of MS.

Of note, while many of the previous seroepidemiological studies analysing EBV seroprevalences in PwMS focused on patients with RRMS, there are considerably fewer data on EBV seroprevalences in PPMS and SPMS [21]. The 100% EBV seroprevalence observed in the present study in patients with PPMS (*n* = 50) and SPMS (*n* = 55) therefore appears to be a relevant observation, suggesting that EBV is associated with all clinical courses of MS.

Others and we have previously proposed that in light of the high EBV seroprevalence in PwMS a negative EBV serology in patients with suspected MS might be a marker for the absence of MS [1, 22, 23, 24]. The finding that all 3 EBV seronegative patients with an ICD10 diagnosis of MS identified in this work had MRI and CSF findings not compatible with MS and did not meet current diagnostic criteria for MS supports this concept. As the EBV seroprevalence in children is lower than that of adults, from a clinical practice point of view, a negative EBV serology as a marker for the absence of MS might be particularly informative in children with suspected MS. Indeed, a negative EBV serology was shown to aid in the differentiation of children with MS and myelin oligodendrocyte antibody associated disease [25, 26, 27]. Our present findings suggest that a negative EBV serology might also aid in the exclusion of MS in adults.

Our data confirm numerous previous reports showing elevated serum levels of EBNA‐1 antibodies in PwMS [7, 8, 9, 10, 11, 12] and make the additional point that such elevations do not occur in patients with 10 different control diseases, again supporting the specificity of the association of EBV and MS. Whereas some previous studies also found elevated VCA antibodies in PwMS as compared to healthy controls [7, 12], the absent elevation of VCA antibodies in PwMS observed in the present study is overall consistent with results of large EBV peptide screens showing that, among all antibodies to EBV, antibodies to EBNA‐1 are most strongly elevated in MS [11, 28].

Only few studies have so far systematically analysed seroprevalences of EBV as compared to that of other common viruses in PwMS [18]. The RISAROLDA approach appeared well‐suited to address this question and showed that seroprevalences of measles, rubella and mumps virus, as well as CMV, HSV and VZV in PwMS were lower than that of EBV and overall reminiscent of the respective seroprevalences in the general population. In a recent study measuring antibodies to EBV and 10 different further common microbes in 50 PwMS, antibodies to EBV were likewise detected in 100% of PwMS, while seroprevalence to 10 further microbes were lower [18]. Together, the results of these studies suggest a unique association of EBV and MS.

We previously showed that, given the 100% EBV seroprevalence in MS, the frequency of an intrathecal production of antibodies to EBV in PwMS is paradoxically low [18, 29, 30]. In particular, the frequency of an intrathecal production of antibodies against EBV is lower than the frequency of intrathecally produced antibodies against other common viruses, whose seroprevalences are lower than that of EBV [18]. We herein analysed this issue by the RISAROLDA approach, which confirmed the paradoxically low frequency of intrathecally produced antibodies to EBV as compared to intrathecally produced antibodies against other common viruses. For a detailed discussion of a possible explanation of this finding, which once more suggests a unique role of EBV in MS, we refer to our previous works [18, 31].

Our study indicates that RISAROLDA is a simple, straightforward and very efficient methodological approach, which permits analysing results of any laboratory parameter obtained during routine diagnostics in large groups of patients. Furthermore, compared to traditional seroepidemiological studies, which frequently involve time‐consuming sample collection and extensive laboratory measurements, RISAROLDA is much less time‐ and cost‐intensive. Thus, RISAROLDA appears to be an attractive method for broad screenings of laboratory data.

Still, RISAROLDA also has a number of limitations. As RISAROLDA is a retrospective approach, the general limitations of retrospective studies apply to RISAROLDA, too. Furthermore, diagnostic categorisation of patients was not performed in a controlled and prospective manner, but relied on ICD10 diagnoses entered in the hospital information system. Although these diagnoses are typically obtained from the patients' discharge letters, the ICD10 diagnoses entered in the hospital information system are not systematically supervised and misdiagnoses can therefore not be completely excluded. Likewise, the digital tools applied in this work did not provide information on the patients' sex or concomitant medication and we were thus unable to include these variables in our analyses. Additionally, RISAROLDA does not provide any information on why treating physicians ordered laboratory tests, for instance, whether infection with a particular virus was suspected. Nevertheless, as the PwMS included in this work did not have the age range in which primary infections with the analysed viruses typically occur, we assume that the majority of antiviral serologies in PwMS were ordered for screening purposes.

Altogether, the present RISAROLDA of a high number of patients underscores a unique association of EBV and MS and suggests that this association is specific for MS as compared to various control diseases. It also suggests that a negative EBV serology represents a marker for the absence of MS. RISAROLDA is a powerful and very efficient tool for *in silico* analysis of real‐world laboratory data, but limitations of this approach have to be borne in mind. While an essential role of EBV in MS appears to be beyond doubt, the underlying mechanisms remain a pressing question.

## Author Contributions

Conceptualization: Mario Rodomonti, Hebun Erdur, Klemens Ruprecht. Formal Analysis: Mario Rodomonti, Klemens Ruprecht. Methodology: Mario Rodomonti, Hebun Erdur, Bettina Eberspächer, Rohat Geran, Klemens Ruprecht. Resources: Bettina Eberspächer. Supervision: Marco Puthenparampil, Paolo Gallo, Klemens Ruprecht. Writing – Original Draft Preparation: Mario Rodomonti, Klemens Ruprecht. Writing – Review and Editing: Florence Pache, Carolin Otto, Patrick Schindler, Bettina Eberspächer, Rohat Geran, Marco Puthenparampil, Paolo Gallo, Brigitte Wildemann, Sven Jarius.

## Conflicts of Interest

F. Pache reports no conflicts of interest. C. Otto is Principal Investigator of the CRC NeuroMac funded by the Deutsche Forschungsgemeinschaft (DFG, German Research Foundation)—Project‐ID 259373024—TRR 167. P. Schindler reports no conflicts of interest. B. Eberspächer reports no conflicts of interest. R. Geran reports no conflicts of interest. M. Puthenparampil reports grants from Almirall, Teva, Sanofi Genzyme, Merck Serono, Biogen Italy, Novartis; consultancy for Novartis, Biogen Italy, Sanofi Genzyme; board membership at Sanofi Genzyme, Novartis, Biogen Italy, and Sandoz; none related to this study. P. Gallo reports grants from Almirall, Teva, Sanofi Genzyme, Merck Serono, Biogen Italy, Novartis, Roche, Bristol Myers Squibb; consultancy for Novartis, Biogen Italy, Sanofi Genzyme, Roche, Bristol Myers Squibb; board membership for Sanofi Genzyme, Novartis, Biogen Italy, Roche, Merck Serono, Bristol Myers Squibb; none related to this study. B. Wildemann reports grants from the Deutsche Forschungsgemeinschaft, German Ministry of Education and Research, Baden‐Württemberg Ministry for Science, Research and Art, Dietmar Hopp Foundation, Klaus Tschira Foundation, grants and personal fees from Merck and Novartis, and personal fees from Alexion, INSTAND e.V., Roche; none related to this study. S. Jarius reports no conflicts of interest. H. Erdur reports no conflicts of interest. K. Ruprecht reports research support from Novartis, Merck Serono, German Ministry of Education and Research, European Union (821283‐2), Stiftung Charité, Guthy‐Jackson Charitable Foundation and Arthur Arnstein Foundation; travel grants from Guthy‐Jackson Charitable Foundation; speaker's honoraria from Virion Serion and Novartis; was a participant in the BIH Clinical Fellow Programme funded by Stiftung Charité.

## Supporting information


**Figure S1:** ene70430‐sup‐0001‐FigureS1.docx.

**Table S1:** ene70430‐sup‐0002‐TableS1.docx.

## Data Availability

The data that support the findings of this study are available on request from the corresponding author. The data are not publicly available due to privacy or ethical restrictions.
