# Supplementary material for: Retrospective In Silico Analysis of Routine Laboratory Data Supports a Specific Association of Epstein–Barr Virus and Multiple Sclerosis
Source: Eur J Neurol. 2025 Nov 12;32(11):e70430. doi: 10.1111/ene.70430 (PMC12612553; doi:10.1111/ene.70430)
Supplement: Supplementary file 1 — Figure S1: ene70430‐sup‐0001‐FigureS1.docx. [file ENE-32-e70430-s002.docx]

**Supplementary Figure 1**


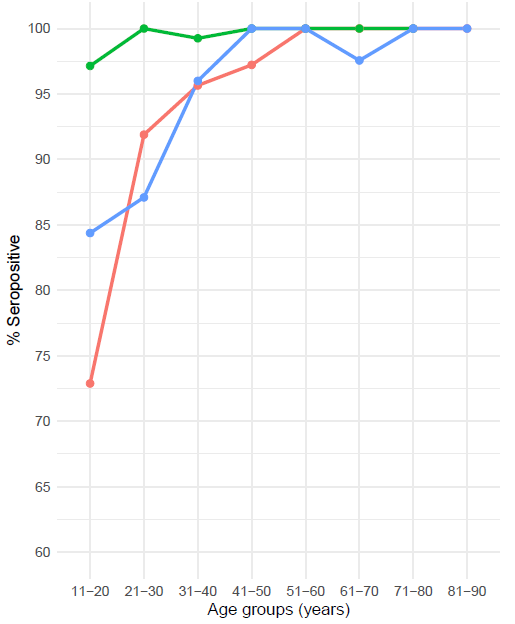


The figure shows the percentage of Epstein-Barr virus (EBV) seropositive patients in the groups of patients with Hodgkin’s lymphoma (n=511, red), autoimmune encephalitides (n=311, blue) and multiple sclerosis (n=492, green) binned into 10-year age intervals. Due to their low numbers, patients <11 years, including a 7-year old EBV seronegative patient with an ICD10 diagnosis of multiple sclerosis (patient #2 in Table 2), were excluded from the analysis. Please note that neither this 7-year old patient nor the two EBV seronegative patients (38-year and 16-year old) with an ICD10 diagnosis of MS included in the analysis (patients #1 and #3 in Table 2) did meet current diagnostic criteria for MS (see Table 2). Please also note that due to their low number patients with Hodgkin’s lymphoma ≥61 years and patients with MS ≥81 years were not included in the analysis.
