# Supplementary material for: Retrospective In Silico Analysis of Routine Laboratory Data Supports a Specific Association of Epstein–Barr Virus and Multiple Sclerosis
Source: Eur J Neurol. 2025 Nov 12;32(11):e70430. doi: 10.1111/ene.70430 (PMC12612553; doi:10.1111/ene.70430)
Supplement: Supplementary file 2 — Table S1: ene70430‐sup‐0002‐TableS1.docx. [file ENE-32-e70430-s001.docx]

**Supplementary Table 1**

**Assays used for determination of antiviral antibodies and antibody indices.**

| Antiviral antibody | Assay used for antibody detection | Order number/code (source) | Method |
| --- | --- | --- | --- |
| EBNA1-IgG | EBV EBNA-1-IgG-ELISA PKS | 126/PKS (Medac) | ELISA |
|  | LIAISON EBNA IgG | REF 310520 (DiaSorin) | CLIA |
| VCA-IgG | Enzygnost Anti-EBV-IgG | OWIS15 (Dade Behring (Siemens)) | ELISA |
|  | LIAISON VCA IgG | REF 310510 (DiaSorin) | CLIA |
| EBV-IgM | Enzygnost Anti-EBV-IgM II | OPC103 (Dade Behring (Siemens)) | ELISA |
|  | LIAISON EBV IgM | REF 310500 (DiaSorin) | CLIA |
| VZV-IgG | Enzygnost Anti-VZV-IgG | OWLT15 (Dade Behring (Siemens)) | ELISA |
|  | LIAISON VZV IgG | REF 310850 (DiaSorin) | CLIA |
| CMV-IgG | CMV-IgG-ELA-Test PKS | 115/PKS (Medac) | ELISA |
|  | Architect CMV IgG | 6C15 (Abbott) | CMIA |
| HSV-IgG | Enzygnost Anti-HSV-IgG | OWMX15 (Dade Behring (Siemens)) | ELISA |
|  | LIAISON HSV-1/2 IgG | REF 310800 (DiaSorin) | CLIA |
| Mumps-IgG | Enzygnost Anti-Parotitis-Virus-IgG | OWLP15 (Dade Behring (Siemens)) | ELISA |
|  | LIAISON Mumps IgG | REF 318840 (DiaSorin) | CLIA |
| Rubella-IgG | ARCHITECT Rubella IgG | 6C17 (Abbott) | CMIA |
|  | Elecsys Rubella IgG | REF 07027770 (Roche) | ECLIA |
| Measles-IgG | Enzygnost Anti-Masern-Virus-IgG | OWLN15 (Dade Behring (Siemens)) | ELISA |
|  | LIAISON Masern IgG | REF 318810 (DiaSorin) | CLIA |
| Antiviral antibody index | Assay used for antibody detection | Order number/code (source) | Method |
| EBV-IgG AI | Enzygnost Anti-EBV-IgG | OWIS15 (Dade Behring (Siemens) | ELISA |
| VZV-IgG AI | Enzygnost Anti-VZV-IgG | OWLT15 (Dade Behring (Siemens) | ELISA |
|  | SERION ELISA classic Varicella Zoster Virus IgG | ESR104G (Virion/Serion) | ELISA |
| CMV-IgG AI | CMV-IgG-ELA-Test PKS | 115/PKS (Medac) | ELISA |
| HSV-IgG AI | Enzygnost Anti-HSV-IgG | OWMX15 (Dade Behring (Siemens) | ELISA |
|  | SERION ELISA classic Herpes Simplex Virus 1/2 IgG | ESR105G (Virion/Serion) | ELISA |
| Mumps-IgG AI | Enzygnost Anti-Parotitis-Virus-IgG | OWLP15 (Dade Behring (Siemens) | ELISA |
|  | SERION ELISA classic Mumps Virus IgG | ESR103G (Virion/Serion) | ELISA |
| Rubella-IgG AI | Enzygnost Anti-Rubella-Virus-IgG | OWBF G15 (Dade Behring (Siemens) | ELISA |
|  | SERION ELISA classic Röteln Virus IgG | ESR129G (Virion/Serion) | ELISA |
| Measles-IgG AI | Enzygnost Anti-Masern-Virus-IgG | OWLN15 (Dade Behring (Siemens) | ELISA |
|  | SERION ELISA classic Masern Virus IgG | ESR102G (Virion/Serion) | ELISA |

AI = antibody index, CLIA = chemiluminescence immunoassay, CMIA = chemiluminescent microparticle immunoassay, CMV = cytomegalovirus, EBNA1 = Epstein-Barr nuclear antigen 1, EBV = Epstein-Barr virus, ECLIA = electrochemiluminescence immunoassay, ELISA = enzyme linked immunosorbent assay, HSV = herpes simplex virus, IgG = immunoglobulin G, IgM = immunoglobulin M, VCA = Epstein-Barr virus viral capsid antigen, VZV = varicella zoster virus
